# Decision criteria for the selection of analytical instruments used in clinical chemistry: VI Techniques for the economic evaluation of automatic analysers

**DOI:** 10.1155/S1463924680000120

**Published:** 1980

**Authors:** Thomas M. Craig

**Affiliations:** E1 du Pont de Nemours & Co Wilmington Delaware 19898 USA


of the laboratory can be determined through experiments of this kind for each proposed structure.

In order to investigate the possibility of including emergency tests in the proposed structures, some further experiments were made. It was shown that the different analysers have different capabilities under these circumstances and that some proposed structures require additional equipment for achieving an acceptable overall performance of the laboratory. This is likely to be expensive.

### Discussion

Simulation experiments are only one phase of the total evaluation project. A final decision must be based on several sets of information, some of which cannot be obtained through simulation experiments. There are several factors of importance not considered in the models used here. These include financial aspects; different reliability aspects for which special studies can be made; factors concerning the possibility of using the equipment in other circumstances than those studied, for example during the night; analytical quality aspects; and man-machine (ergonomic or environmental) aspects.

The models are applicable to most clinical laboratory systems. They can be adapted to local circumstances through the adjustment of input parameters. Results from stimulation experiments are, however, mostly specific to each studied laboratory system. For example significant variations in the request profiles can have a considerable influence on the results. The models are flexible in that they describe the laboratory system as a whole and can therefore be used for studies of different aspects of problems connected with the planning process.

Other problems can be introduced when evaluating the results from the simulation experiments. Thus a change in the layout of the specimen reception area can result in a changed reception pattern and this is particularly important when multichannel analysers are involved. Under these circumstances the conditions of evaluation are changed and the results from the simulation experiments are no longer valid. Such effects can be difficult to predict, and a sensitivity analysis of the result with respect to such variations must be made.

Another important factor not included in these studies is the evaluation with respect to the external effectiveness of the laboratory, i.e. the "medical benefit" of the report from the laboratory investigation. Some multichannel analysers produce reports in the form of test-profiles, where tests are reported which are not necessarily requested. The medical benefit of such reports is difficult to evaluate but has been extensively discussed elsewhere.

Techniques and methods for simulation studies with respect to external effectiveness are being developed [5].

REFERENCES

[1] Sandblad, B., Systems analysis and simulation of health care laboratory systems - methodology and models. UPTEC 77 13R (1977), Institute of Technology, Uppsala University. To be published in *Computer Programs in Biomedicine*.

[2] W Schneider and S Bentsson eds.: The application of computer techniques in health care. *Computer Programs in Biomedicine*, (1976) 5(3) 169-250.

[3] Sandblad, B., Modelling and simulation for planning and cost/benefit analysis of health care service systems. *Medical Informatics* (1976) 1, (1), 27-37.

[4] Sandblad, B., Hellsing, K. and de Verdier, C. H. The use of multichannel analytical equipment in clinical chemistry – a simulation study. UPTEC 77 16R (1977), Institute of Technology, Uppsala University.

[5] Bengtsson, S., Sandblad, B., Schneider, W. and Spencer, W. A. Effectiveness studies in connection with health care service functions. IFIP working conference on evaluation of efficacy of medical action. Bordeaux 14-19 May 1979.

ACKNOWLEDGEMENTS

This paper was prepared in collaboration with Uppsala University Data Centre and the Department of Clinical Chemistry, Uppsala University Hospital.

### Decision criteria for the selection of analytical instruments used in clinical chemistry

### VI Techniques for the economic evaluation of automatic analysers

**Thomas M. Craig**

*EI du Pont de Nemours & Co, Wilmington, Delaware 19898, USA*

The financial evaluation of automation is a three-step process. Firstly, it is necessary to determine present costs in the laboratory and thus provide a base with which possible alternatives can be compared. Secondly, computation of the total cost of each alternative is required, including both initial acquisition cost and operating costs. Thirdly, the cost/benefit assessments of the alternatives need be compared in the light of their ability to satisfy specific requirements.

The current laboratory costs may be considered under two headings - direct and indirect; of these, only the former is relevant to decision making on the installation of an automatic analyser. These direct costs, shown in Figure 1, include coverage of supplies, labour, reagents (including wastage), standards, controls, and any repeat or duplicate measurements required. In most cases indirect costs, such as expenditure on supervision and overheads will not change no matter which analyser is selected.

The Hospital Administrative Services Group of the American Hospital Association publishes a survey of the direct costs in hospitals. It is based on data from 1,800 hospitals. The average direct cost per test in any laboratory is determined by dividing the total direct cost by the number of tests run. As can be seen from Figure 2 (which shows the results from the last survey collecting data in the direct cost/test format which was conducted in 1976), costs varied significantly according to hospital bed size.

Batch size (i.e. the number of tests processed at once) is a significant factor which must be considered in determining current costs. A breakdown of fixed cost/variable cost illustrates the reason for this. For example, in the case of an automatic analyser, fixed cost includes start-up, shutdown and preparation of standard calibration curves. Variable cost includes the reagents, the variable portion of labour and the variable proportion of tests used for quality control purposes, (in the case of a Technicon AutoAnalyser, typically one control is processed with every nine tests). Thus, the fewer the number of tests the greater the impact the fixed cost has on the cost per test. The effect of batch size on the cost per test is shown in Figure 3.

After determining the current costs of carrying out the test load in question, the second and third stages of the analysis relate to cost evaluation of the instruments themselves.

In determining whether the workload justifies the installation of a particular automatic instrument it is important to find the breakeven estimate of the number of tests. This aspect is illustrated in Figure 4. The cost-volume relationship may be curvilinear, a step function, or close to a straight line. The important information obtained is the crossover point of the two lines. The workload at this point is referred to as the 'critical batch size.' If the current load is below this point, then costs will be greater after the new equipment is installed. If it is above, the proposed instrument would give greater cost effectiveness.

The level of workload required to justify the proposed automation is determined using the equation shown in Figure 5. Reading from left to right, the equation shows the average present cost per test for the tests in question times X (the annual volume needed before the alternative is justified); the annual depreciation in value of the alternative (fixed); the average consumable cost (variable); the cost of other supplies required to operate the alternative automation (variable); the cost of quality control (a portion of which will be fixed

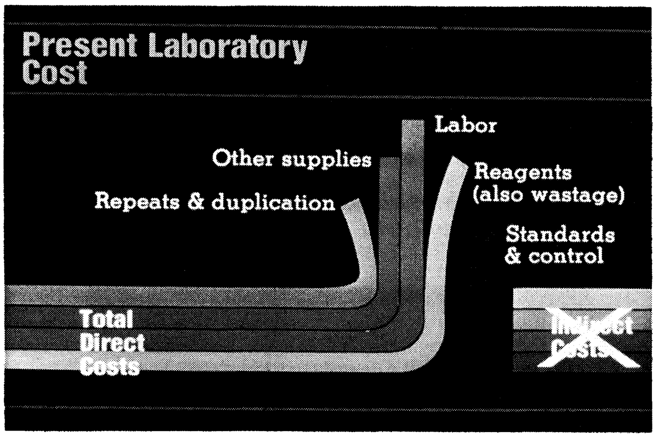

*Figure 1.*

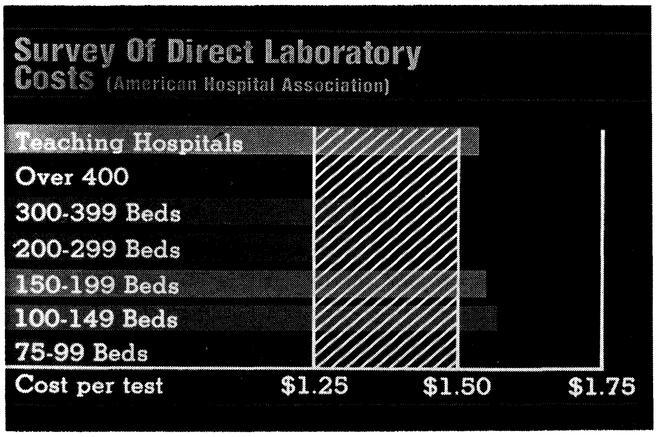

*Figure 2.*

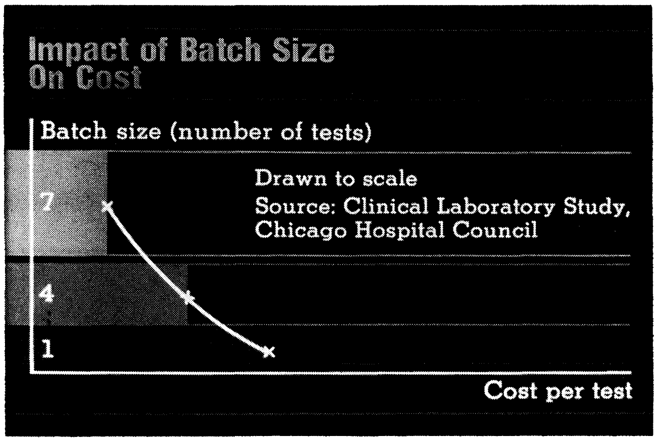

*Figure 3.*

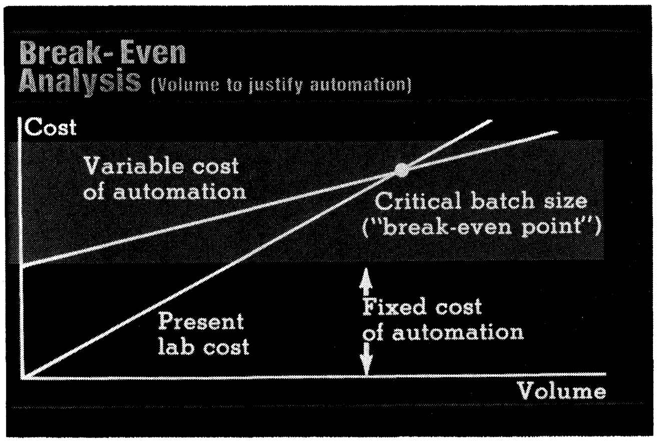

*Figure 4.*

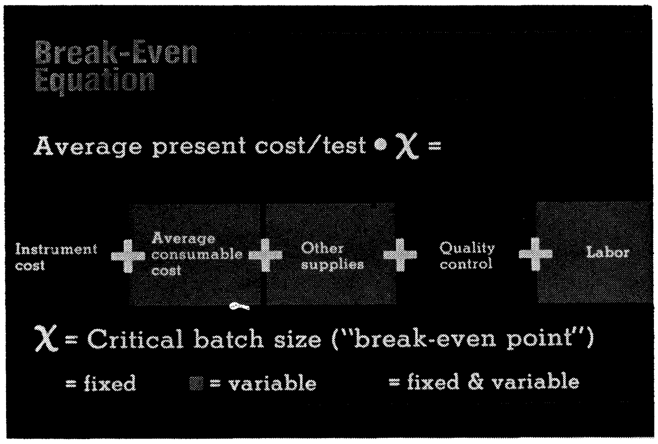

*Figure 5.*

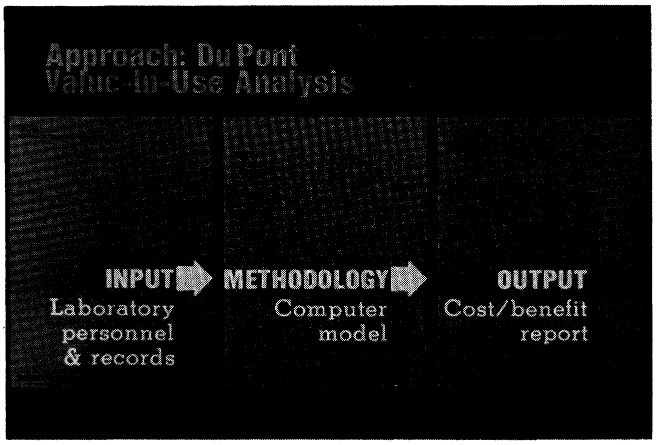

*Figure 6.*

and a portion variable), and labour (considered here as a variable). Solving the equation for X determines the economic breakeven point.

### An example of the practical use of cost analysis in evaluating automation

The Du Pont Value-In-Use Analysis, an approach developed by the author a number of years ago, can be used to illustrate an application of the above. It has been applied to a large number of different situations in clinical laboratories throughout the United States and in Europe. In the general scheme shown in Figure 6, the input, based on interviews of laboratory personnel and laboratory records, is fed into a computer model containing 20 different cost equations. The model was based upon information obtained from several independent sources including the College of American Pathologists' Workload Recording Method for Clinical Laboratories; The Canadian Schedule of Unit Values for Clinical Laboratories - a joint study by the Canadian Association of Pathologists and the Dominion Bureau of Statistics; and The Clinical Laboratory Study by the Chicago Hospital Council. An 11-page cost/benefit analysis report is automatically prepared from the data submitted.

The same overall approach has been applied uniformly throughout the world, since the model has the flexibility to use cost and time factors specific to a given laboratory or a given area.

The Value-In-Use Analysis programme calculates the direct cost comparison between present laboratory costs and the cost of performing the same tests on this Du Pont analyser. It then prints out the results. The volume, given the laboratory's present costs, which the laboratory would have to run to break-even on the Du Pont system is then calculated and compared to the actual volume. Also included in the output is a revenue generating statement (if appropriate) which can be used for hospital priority considerations. The text of the report is available in English, French or German, and the costs are presented in the appropriate currency.

In addition to direct cost comparisons and economic breakeven point analyses, there are other techniques affecting automation evaluation which are often useful. These include return on investment, payback period, present value index and discounted cash flow rate of return.

Many other factors influence the results, the most critical of which is perhaps instrument depreciation, which in turn relates to the estimated useful life of laboratory equipment.

Return on investment is a technique for calculating the amount of money one earns per unit of investment. The automatic analyser showing the highest return is the preferred system selected using this criteria.

Another technique for evaluating instrumentation is the payback period calculation. This is particularly useful in a hospital where cash flow is a prime consideration. Its calculation in cash-tight institutions would certainly assist the administrator in evaluating any proposal and compare the benefits with other proposals from different departments within the organisation. Payback period addresses one of the administrator's major concerns – how long will the particular piece of automation take to pay for itself? The payback period is defined as the time period required to generate sufficient cash flow to cover the cash outflow required to purchase the instrument. The calculation is a fairly straightforward procedure.

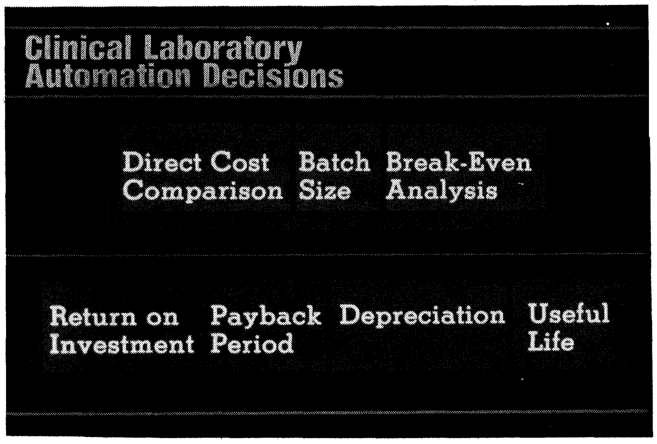

*Figure 7.*

Two other parameters also need to be considered - present value index and discounted cash flow. The present value index is defined as the relationship of the present value of cash inflow to the present value of cash outflow. To assist in the determination, present value tables are available in standard texts to calculate the index. The discounted cash flow rate of return is defined as the rate which will make the cash inflow equal to the cash outflow. Both can be calculated. These factors may be used to compare the various automation alternatives under consideration. Both techniques have the considerable advantage of being independent of inflation. However, they probably provide a degree of sophistication which is not normally required, and often less complex approaches will suffice.

Two basic types of depreciation need to be considered, that is accelerated and straight line. The two most common accelerated approaches to the measurement of depreciation are known as sum of the year's digits and the double declining balance. However some medical insurance organisations do not accept considerations which include accelerated depreciation and the best approach is probably the conservative straight line calculation. In this approach the acquisition cost of the automation is simply divided by its useful life. The useful life assigned to automatic analysers is determined by the assessment of the life of the instrument relating to both physical deterioration and the product obsolescence.

Laboratories operate in an environment of limited resources which creates a driving force to ensure that any capital resources allocated are well placed. Figure 7 summarises the techniques and associated parameters utilised in cost analysis to help ensure that the resources for laboratory automation are applied in a cost-effective manner.

